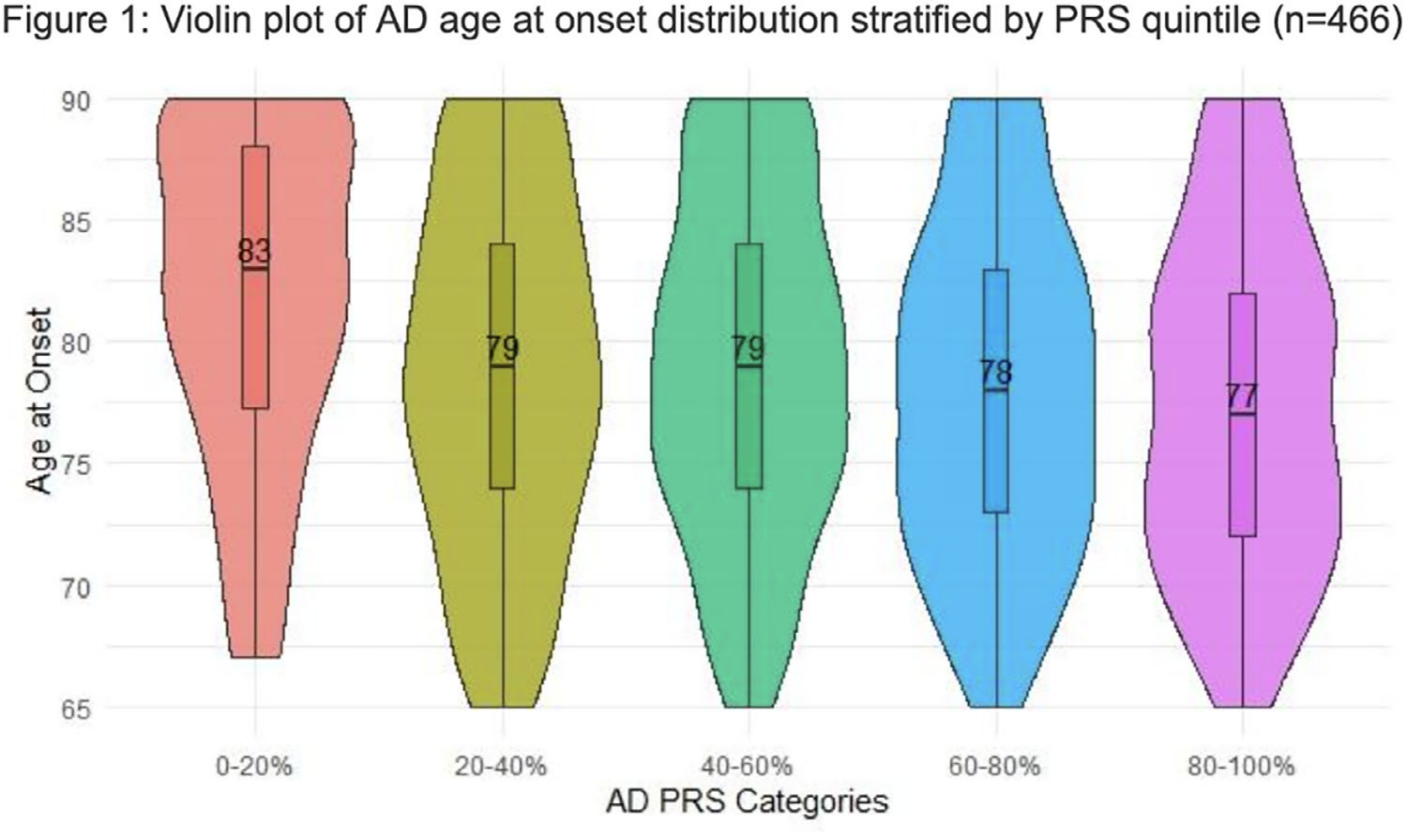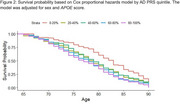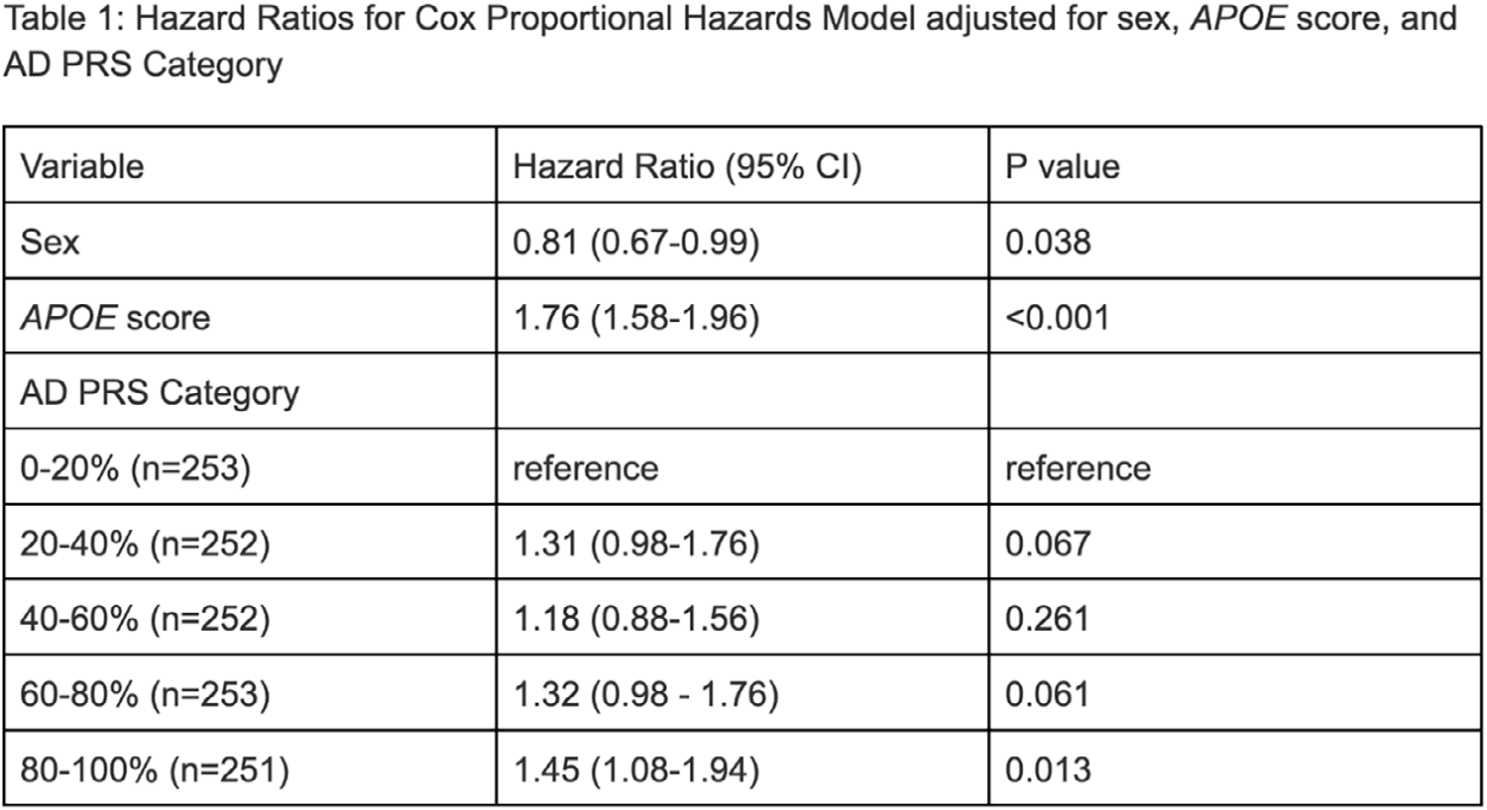# Polygenic Risk Scores and Age at Onset in Late‐Onset Alzheimer's Disease: Insights from a Multi‐Ancestry Cohort

**DOI:** 10.1002/alz70860_104110

**Published:** 2025-12-23

**Authors:** Jovana Jovanovska, Corinne D. Engelman

**Affiliations:** ^1^ University of Wisconsin‐Madison, School of Medicine and Public Health, Madison, WI, USA; ^2^ University of Wisconsin‐Madison School of Medicine and Public Health, Madison, WI, USA

## Abstract

**Background:**

Neuropathological changes in late‐onset Alzheimer's disease (LOAD) precede symptoms by years, underscoring the need for early detection. While the *APOE* ε4 allele is the strongest genetic risk factor, it does not fully explain LOAD risk. Polygenic risk scores (PRS), which aggregate multiple genetic variants, show promise in predicting Alzheimer's risk and age at onset (AAO). However, most PRS models are based on European‐ancestry populations, limiting applicability to other ancestries. Furthermore, the relationship between PRS and AAO remains unclear, particularly across diverse populations. This study evaluates whether higher PRS is associated with earlier AAO in a multi‐ancestry cohort from the Alzheimer's Disease Sequencing Project (ADSP).

**Method:**

Data were collected from three ADSP cohorts, comprising 1261 participants (466 LOAD cases, 795 controls) of European, African American, and Hispanic ancestry. PRS scores were derived from 40 variants identified in a multi‐ancestry genome‐wide association study (GWAS). Participants were categorized into quintiles based on their PRS score. A Cox proportional hazards model, with age as the timescale, was used to estimate hazard ratios (HRs), adjusting for sex and *APOE* score.

**Result:**

Higher PRS was associated with earlier AAO. Participants in the highest PRS quintile (80–100%) had a 45% higher hazard of developing LOAD compared to the lowest quintile (0–20%) (HR = 1.45, 95% CI: 1.08–1.94, *p* = 0.013). *APOE* score was strongly predictive of LOAD (HR = 1.76, 95% CI: 1.58–1.96, *p* < 0.001), while female sex had a modest protective effect (HR = 0.81, 95% CI: 0.67–0.99, *p* = 0.038). Violin plots showed a stepwise decline in median AAO with increasing PRS categories, from 83 years in the lowest quintile to 77 in the highest.

**Conclusion:**

Higher PRS is associated with earlier AAO, supporting its utility in identifying high‐risk individuals. PRS models based on the first multi‐ancestry GWAS represent progress but could improve with larger datasets. Incorporating genetic, environmental, and lifestyle factors may further enhance the accuracy of PRS in diverse populations. Analyses are underway with the new ADSP Release 5.